# Benign mosaic chromosomal structural variants across generations: evidence for a developmental correction mechanism from clinical and computational models

**DOI:** 10.3389/fgene.2025.1710280

**Published:** 2025-11-20

**Authors:** Jiayu Ruan, Qinhao Song, Yue Hu, Xiaodan Liu, Suping Li, Jianjun Zhu, Li Yang

**Affiliations:** 1 School of Life Sciences and Technology, Tongji University, Shanghai, China; 2 Prenatal Diagnosis Center, Jiaxing Maternity and Child Health Care Hospital, Jiaxing, Zhejiang, China

**Keywords:** chromosomal mosaicism, supernumerary marker chromosome (SMC), structural variant chromosome (SV), embryonic self-correction, computational modeling, Shepherd Mechanism

## Abstract

**Objective:**

This study investigates the intergenerational transmission of benign mosaic supernumerary marker chromosomes or structural variant chromosomes (SMCs/SVs) and explores the developmental mechanisms that maintain non-pathogenic mosaic levels across generations. While chromosomal mosaicism is widely recognized in reproductive genetics, most previous work has focused on pathogenic outcomes. Here, we highlight an underexplored phenomenon of non-pathogenic SMCs/SVs mosaicism and propose a developmental selection model that may explain its stable inheritance.

**Methods:**

We describe a rare father–offspring pair carrying a mosaic SV at chromosome 11p11, both phenotypically normal. Karyotyping and SNP-array analyses were performed on parental blood, amniotic fluid, and cord blood. A systematic literature review identified 35 additional families with benign parent–child SMCs/SVs mosaicism. To probe potential regulatory mechanisms, four complementary computational approaches including agent-based simulation, logistic regression, Bayesian inference, and Markov chain modeling were applied to evaluate the developmental selection dynamics.

**Results:**

The father exhibited a 57% SV mosaic ratio, while the offspring showed comparable or slightly reduced ratios (38%–45%). Literature analysis revealed consistent patterns of equal or lower mosaicism in offspring across diverse SMCs types, suggesting that transmission occurs within a constrained, non-pathogenic range. Computational modeling demonstrated that even mild negative selection during blastocyst development could reproduce these retention trends, supporting a developmental selection mechanism that limits SMCs/SVs-positive cells to a harmless threshold.

**Conclusion:**

These findings provide convergent clinical and computational evidence that early human embryos may employ a self-correction mechanism to regulate benign SMCs/SVs mosaicism. We propose a developmental “Shepherd Mechanism,” whereby mosaic cells are selectively eliminated until a safe equilibrium is reached, ensuring viable yet non-pathogenic inheritance. This work introduces a conceptual framework for understanding naturally tolerated chromosomal variation and offers theoretical guidance for prenatal genetic counseling and embryo selection strategies in assisted reproduction.

## 1 Introduction

Chromosomal mosaicism refers to the coexistence of two or more cell populations with different chromosomal complements within a single individual (Militaru et al., 2024; Spinner and Conlin, 2014). Chromosomal mosaicism typically arises from random mutations or mitotic errors during the early embryonic cell divisions following fertilization. Proposed mechanisms include mitotic nondisjunction, chromosome lagging, or trisomy rescue events (Campbell et al., 2015; McCoy, 2017; McCoy et al., 2015; Grati, 2014). Conventional methods for chromosome analysis, primarily G-banding and C-banding karyotyping, are limited by their intrinsic resolution, making it challenging to detect low-level mosaicism or subtle structural chromosomal abnormalities (Xue et al., 2020). Consequently, some aberrations may go undetected in clinical practice (Grati, 2014; Popovic et al., 2020; Eggenhuizen et al., 2021). In recent years, high-resolution molecular techniques such as fluorescence *in situ* hybridization (FISH), comparative genomic hybridization (CGH), single nucleotide polymorphism arrays (SNP-array) (Zhou et al., 2023; Li et al., 2022), digital PCR (dPCR) (Dube et al., 2008) and next-generation sequencing (NGS) (Fan et al., 2010) have been widely adopted. These technologies greatly enhance both the detection rate of chromosomal mosaicism and its precise subclassification, thereby improving accuracy and efficacy in prenatal diagnosis.

Supernumerary marker chromosomes (SMCs), as a special type of chromosomal abnormality, are typically smaller than chromosome 20 and often lack definitive banding patterns, posing substantial challenges for traditional karyotyping in determining their chromosomal origin (Liehr and Al-Rikabi, 2019; Gosden et al., 1988). Common SMCs configurations include ring chromosomes (r), inverted duplicated chromosomes (inv dup), and centromeric mini-chromosomes (min) (Xue et al., 2020; Hu and Kong, 2023; Yao et al., 2021). Epidemiological data indicate that the occurrence rate of SMCs in live births is about 0.043% (Lebedev et al., 2021), with approximately 70% of carriers displaying no obvious clinical abnormalities, whereas the remaining 30% exhibit varying degrees of phenotypic anomalies (Liehr and Weise, 2007; Liehr et al., 2023). In recent years, researchers such as the Liehr group have employed advanced technologies, including FISH, array CGH, and SNP-array, to deeply investigate the genomic structure of SMCs, the breakpoints involved, and the distinction between dosage-sensitive and dosage-insensitive chromosomal regions (Liehr et al., 2023; Manvelyan et al., 2008). The specialized SMC database established by the Liehr team consolidates a vast array of SMCs cases involving chromosomes 1–22 as well as the sex chromosomes. These studies have not only expanded our understanding of the diverse phenotypic presentations of SMCs but also provided valuable data concerning mosaic formation during vertical transmission within families (Liehr et al., 2009; Liehr et al., 2021; Liehr et al., 2011). Although classical SMCs represent supernumerary chromosomal fragments, recent cytogenomic studies have suggested that small structural variants (SVs), particularly insertional or duplicated segments involving pericentromeric regions, may share highly similar formation and retention mechanisms with inv dup-type SMCs (Liehr et al., 2023; Rodríguez, 2023). Both SMCs and these SVs arise from local duplication or rearrangement events during early embryonic divisions, potentially persisting as benign mosaic populations regulated by developmental selection (Liehr et al., 2023; Joksic et al., 2023).

These observations suggest that understanding the developmental dynamics governing the maintenance of benign mosaic states is essential to elucidate their intergenerational stability. Despite substantial advancements in SMCs/SVs detection technologies, classification methods, and associations with clinical phenotypes, our understanding of the precise mechanisms underlying mosaic formation remains limited (Grati, 2014; Zhang and Zheng, 2024). This gap is especially evident in clinical prenatal settings, where it remains challenging to provide accurate genetic counseling based solely on mosaic findings (Liehr and Al-Rikabi, 2019; Levy et al., 2021). One principal obstacle stems from the extremely brief and complex stages from fertilization to blastocyst formation, during which subtle chromosomal alterations can substantially influence embryonic development but remain difficult to observe with current experimental methods (Zhang and Zheng, 2024; Benn, 1998; Ma et al., 2017).

Clinically, chromosomal mosaicism not only potentially affects fetal growth and development but may also impact placental function. Confined placental mosaicism (CPM) has been linked to adverse pregnancy outcomes, including fetal growth restriction or preterm births (Militaru et al., 2024; Eggenhuizen et al., 2021; Hu and Kong, 2023; Joksic et al., 2023). Moreover, with the widespread adoption of non-invasive prenatal testing (NIPT), placental mosaicism may result in false-positive or indeterminate findings, further complicating clinical decision making (Liehr and Al-Rikabi, 2019; Grati et al., 2017).

In this study, we integrate clinical case evidence with literature-based data to elucidate the mechanisms of chromosomal mosaicism, particularly SMCs/SVs mosaicism and propose a novel theoretical model that more accurately describes mosaic formation in intergenerational transmission. To address the inability to directly observe early embryonic correction mechanisms, we constructed four computational frameworks including agent-based simulation, logistic regression, Bayesian inference, and Markov chain modeling, each offering complementary perspectives on the regulation of SMC/SV + cells during blastocyst formation. This modeling strategy allows us to investigate the plausibility, stability, and statistical robustness of the proposed “Shepherd Mechanism” under realistic biological constraints. By incorporating both empirical clinical observations and quantitative simulations, this work seeks to bridge a critical knowledge gap and provide a reproducible systems-level framework for understanding non-pathogenic mosaic retention during human embryogenesis. Such a framework not only advances our theoretical understanding of mosaicism but also promises to improve prenatal diagnostics and genetic counseling by offering more specific and reliable guidance, thereby enhancing both the quality and precision of clinical care.

## 2 Methods

### 2.1 Subjects

In 2024, a 27-year-old pregnant woman (G3P0) was referred to the Jiaxing Maternal and Child Health Hospital due to her husband’s mosaic chromosomal abnormality. Her menstrual cycles were regular, and this pregnancy occurred naturally; this was her third pregnancy, following two previous biochemical pregnancies that did not progress beyond early gestation. At 18 weeks of gestation, she underwent prenatal consultation with fetal medicine specialists. After being fully informed of the potential benefits and limitations of diagnostic testing and providing written informed consent, she underwent amniocentesis for fetal karyotyping and SNP-array analysis. Because of inconsistent findings between the fetal karyotype and SNP-array results, serial ultrasound examinations were performed in the later stages of pregnancy, all of which showed no fetal abnormalities. The patient opted to continue the pregnancy and agreed to postnatal cord blood karyotyping as well as placental karyotype verification. The verification protocol was approved by the Ethics Committee of Jiaxing Maternal and Child Health Hospital (Approval No.: 2024-Y-66). She delivered a healthy female infant (birth weight, 2910 g), with normal Apgar scores; the infant has since been followed up regularly with no developmental anomalies noted to date.

### 2.2 Methods

#### 2.2.1 Experimental methods for detecting paternal mosaic transmission

Peripheral blood from the father and cord blood from the newborn were cultured for 72 h in peripheral blood-specific medium. Following colchicine treatment, hypotonic shock, fixation, and standard G-banding, 100 metaphases were screened, and 15 karyotypes were analyzed in detail. SNP-array analysis was performed on the father’s peripheral blood using the Affymetrix CytoScan 750K platform. Genomic DNA was extracted using the QIAamp DNA Blood Mini Kit (Thermo Fisher Scientific), and the assay was conducted according to the manufacturer’s instructions. Data were processed with ChAS 4.3 software and interpreted following ClinGen (Riggs et al., 2020) and ACMG guidelines (Richards et al., 2015).

Amniotic fluid was collected via ultrasound-guided amniocentesis at 18 gestational weeks. Twenty milliliters were used for G-banded karyotyping, and 10 mL for SNP-array analysis. Genomic DNA from amniotic fluid was extracted and hybridized following the same procedure as above. Standard cytogenetic protocols were applied for chromosome preparation, including 7-day culture, colchicine harvest, hypotonic treatment, methanol/acetic acid fixation, slide preparation, and Giemsa staining. Metaphases were scanned using a Leica automated imaging system. Twenty cell clones were selected, with 2–3 karyotypes analyzed per clone.

#### 2.2.2 Literature review methods

A comprehensive literature search was conducted in the PubMed, Web of Science, and Scopus databases, without any publication date restrictions but limited to English-language articles. A combination of free-text terms and controlled vocabulary was employed, focusing on the keywords “Supernumerary marker chromosome (SMC),” “mosaicism,” “prenatal diagnosis,” and “inheritance.” The literature search concluded on 1 April 2025, with periodic updates planned as needed. Eligible studies were required to explicitly document the presence of parental SMCs transmitted to offspring, with the offspring showing mosaicism. Articles reporting clinical phenotypes without specifying the genetic origin of the SMCs were excluded. From the studies meeting inclusion criteria, we systematically extracted data on the parental and offspring SMC mosaic ratios, SMC types, chromosomal regions involved, clinical presentations, and family inheritance patterns.

#### 2.2.3 Computational modeling of mosaic retention

Four computational modeling strategies were applied to simulate the developmental regulation of SMC/SV + mosaicism during early embryogenesis.

##### 2.2.3.1 Agent-based simulation

An agent-based model was constructed to represent individual SMC/SV+ and SMC/SV− cells undergoing proliferation under selective constraints. Cells were spatially allocated to the inner cell mass (ICM) or trophectoderm (TE) compartments. The model incorporated mild selection pressure against SMC/SV + cells. Spatial segregation and mosaic ratio retention were recorded across simulated developmental cycles.

##### 2.2.3.2 Logistic regression modeling

Logistic regression was used to fit mosaic retention curves based on a dataset of 35 parent-offspring SMCs/SVs mosaicism cases. The model was structured to quantify the relationship between parental mosaic levels and those observed in offspring, enabling the detection of non-linear deviations from a 1:1 transmission pattern.

##### 2.2.3.3 Bayesian inference

A Bayesian beta-binomial framework was implemented to assess whether mosaic reduction in offspring deviated from random expectation. Posterior probabilities were calculated for the hypothesis that offspring mosaic ratios are systematically lower than parental levels, based on the observed data distribution.

##### 2.2.3.4 Markov chain analysis

A discrete-time Markov chain was built to simulate fate transitions of SMC/SV + cells across embryonic development stages. Defined states included apoptosis, TE allocation, and ICM retention. Transition probabilities were iteratively applied to estimate equilibrium distributions and the expected proportion of SMC/SV + cells within the ICM.

#### 2.2.4 Comparative evaluation of modeling approaches

The four modeling strategies were compared using a structured evaluation framework adapted from Hodzic and Sindi (2020, Developmental Biology). Each model was assessed across four dimensions: mechanistic interpretability, spatial resolution, predictive fit to empirical trends, and statistical inference strength. Agent-based simulation was rated highest in mechanistic and spatial dimensions due to its capacity to simulate cell-level behavior and tissue compartmentalization. Logistic regression achieved the best performance in trend-fitting across empirical mosaic ratios. Bayesian inference provided the strongest statistical confidence through posterior distribution analysis. Markov chain modeling contributed moderate interpretability and spatial inference but excelled in illustrating dynamic cell fate transitions. Qualitative scores were assigned (Low, Moderate, High) across each axis to inform integrated assessment.

## 3 Results

### 3.1 Paternal genetic testing results

**FIGURE 1:**
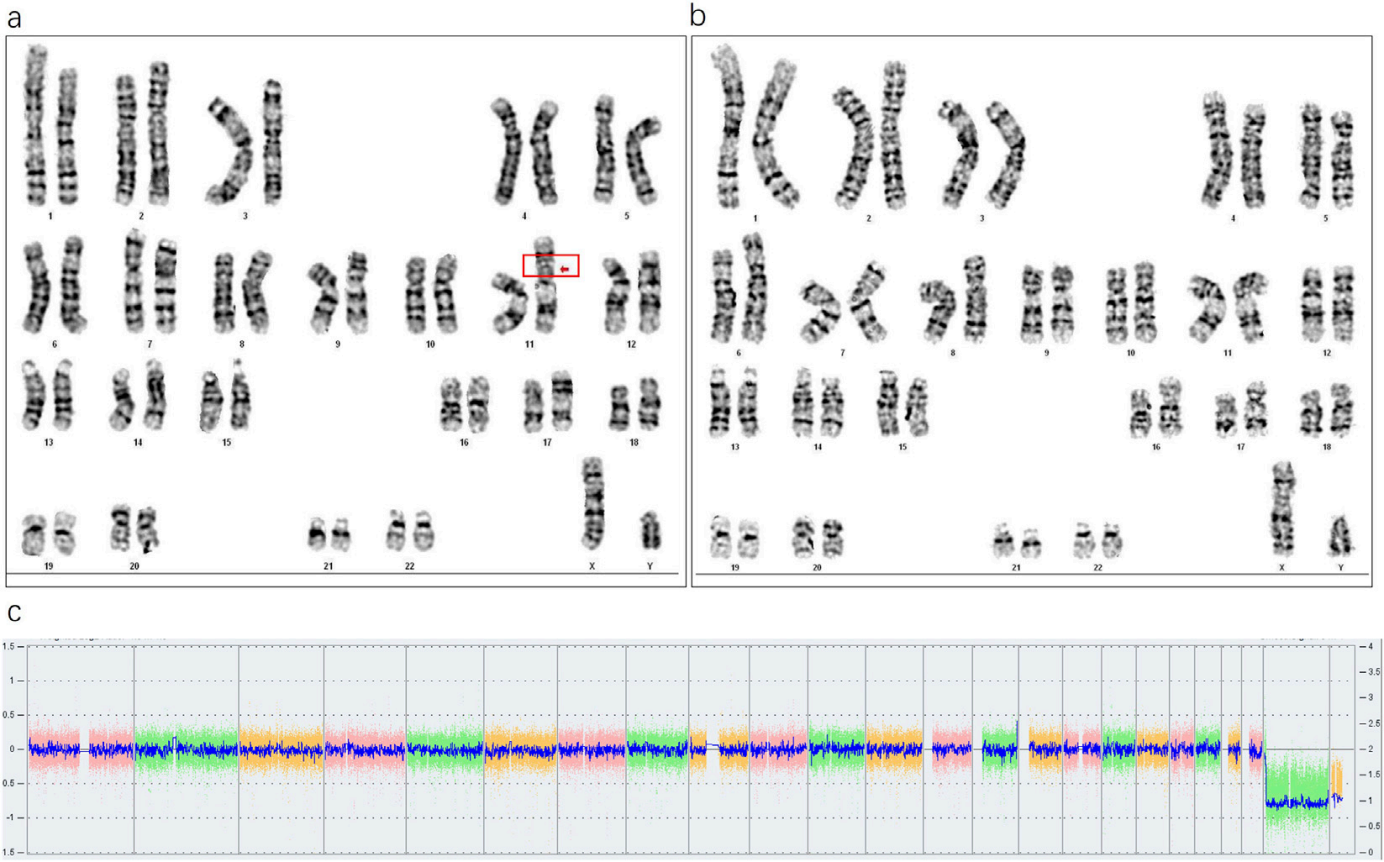
**(a)** The red box and arrow indicate the inserted fragment (57%, 17/30). **(b)** Normal cell karyotype. **(c)** SNP-array analysis of the paternal peripheral blood, showing no abnormalities.

Chromosomal karyotyping of the father’s peripheral blood cells revealed a mosaic pattern, described as 46,XY,ins (11;?) (p11;?) (Yao et al., 2021)/46,XY (Fan et al., 2010). Specifically, an unknown SV fragment was inserted into the short arm of chromosome 11 at band p11 (Figure 1a), with a mosaic ratio of approximately 57% (Figures 1a,b). Further single nucleotide polymorphism array (SNP-array) analysis of the father’s peripheral blood indicated no detectable copy number variations (CNVs) or other genomic abnormalities (Figure 1c). SNP-arrays have limited probe coverage for benign or unannotated genomic regions, which may lead to false negatives in non-pathogenic insertions.

### 3.2 Fetal karyotype identification

**FIGURE 2:**
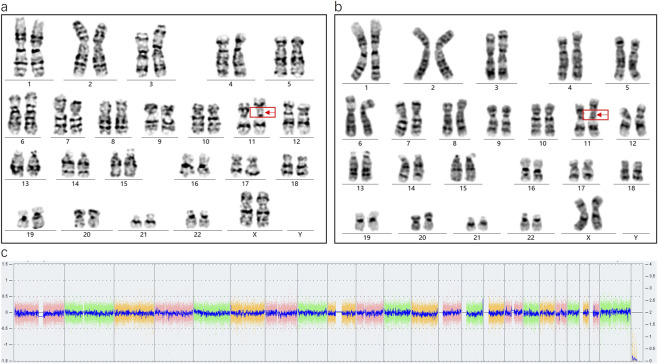
**(a)** Abnormal karyotype in fetal amniotic fluid (38%, 13/34), **(b)** Abnormal karyotype in neonatal cord blood (45%, 45/99), **(c)** SNP-array results of the amniotic fluid sample showing no abnormalities.

**FIGURE 3:**
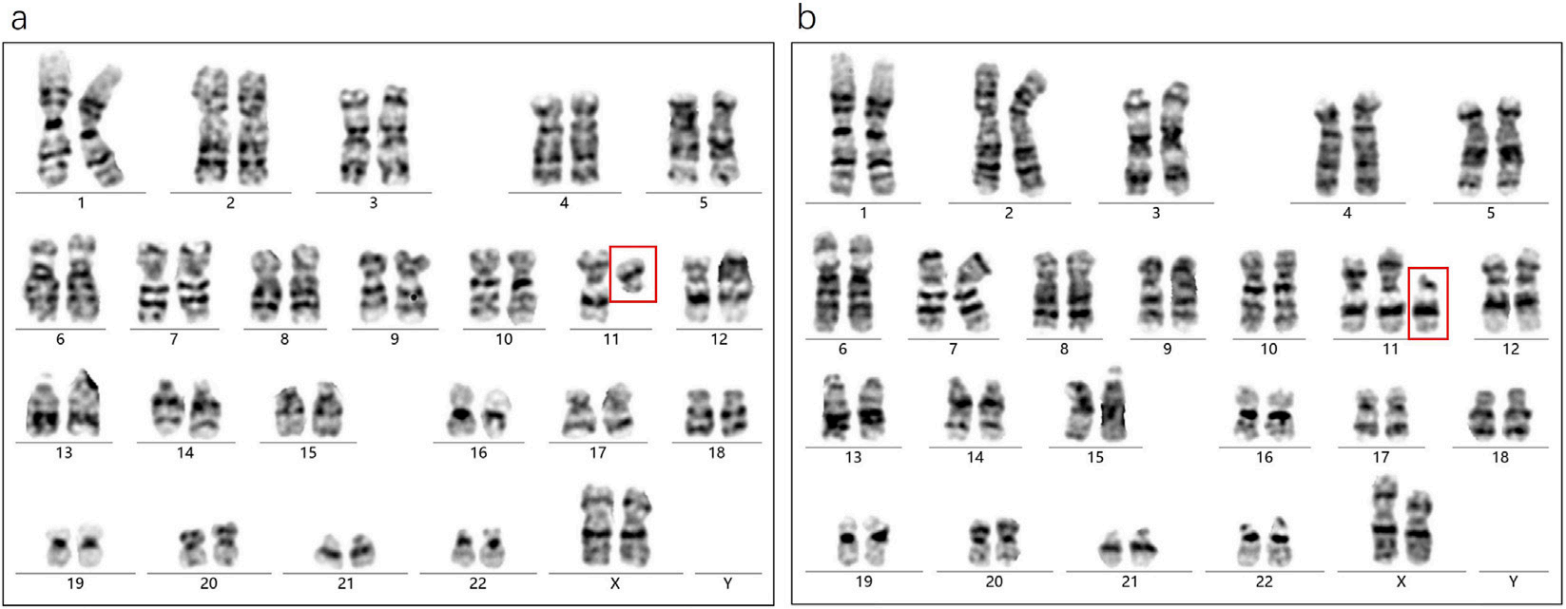
**(a)** The amniotic fluid sample shows an extremely low-level mosaicism involving a deletion on the long arm of chromosome 11 (8.8%, 3/34). **(b)** The cord blood sample shows an extremely low-level mosaicism involving a duplication on the long arm of chromosome 11 (3%, 3/99). Both anomalies share breakpoints at 11p11.

Karyotype analysis revealed a mosaic pattern in both prenatal and postnatal samples. The fetal karyotype from amniotic fluid (AF) was 46,XX,ins (11;?) (p11;?) (Fan et al., 2010)/46,XX (Hu and Kong, 2023), while the postnatal karyotype from cord blood (CB) was 46,XX,ins (11;?) (p11;?) (Karaman et al., 2006)/46,XX (Sun et al., 2017). In this offspring, a SV-1 was identified prenatally with a mosaic ratio of 38% in the AF sample, while a SV-2 was detected postnatally with a mosaic ratio of 45% in the CB sample (Figures 2a,b). Further SNP-array analysis of the fetal amniotic fluid showed no significant copy number variations (CNVs) or other genomic abnormalities (Figure 2c). Comparative chromosome banding results showed that the fetal SV-1 and SV-2 were identical to the paternal SV fragment, supporting that the mosaic structural variant originated in the father and was transmitted to the offspring in a mosaic form. In addition, a very low-level mosaicism involving deletions in the short arm and/or duplications in the long arm of chromosome 11 was detected in both the prenatal amniotic fluid (8.8%) and postnatal cord blood samples (3%) (Figures 3a,b).

### 3.3 Analysis of the mechanism of intergenerational mosaic inheritance

#### 3.3.1 Genetic pattern in the present case

**FIGURE 4:**
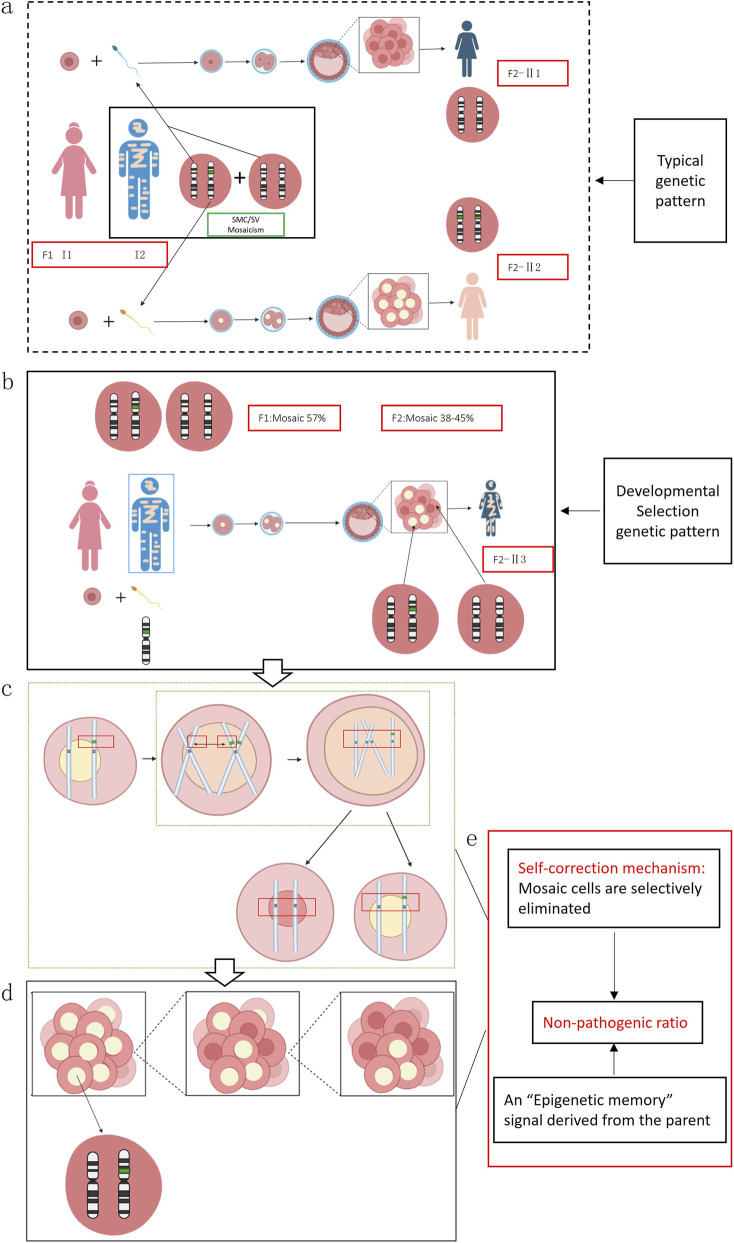
**(a)** In this scenario, an oocyte is fertilized by a sperm carrying a normal chromosome 11, which should theoretically give rise to offspring with a normal karyotype (II1); alternatively, if the sperm carries a chromosome 11 with an inserted segmental variant SV (green fragment), fertilization would theoretically result in a non-mosaic abnormal karyotype, designated as 46,XX,ins (11;?)(p11;?) (II2). **(b)** An oocyte from individual I1 is fertilized by a sperm from individual I2 that carries chromosome 11 containing an unknown inserted fragment, thereby producing individual (II3). **(c)** During mitotic divisions of the inner cell mass at the blastocyst stage, homologous recombination (sister-chromatid short-arm exchange) induces a chromosomal break and leads to loss of the SV fragment. **(d)** After the sister chromatid harboring the SV is removed and the remaining chromatid pairs with the originally normal sister chromatid, some cells in the inner cell mass revert to a normal karyotype, causing the embryo produced in the preceding steps to ultimately develop as a mosaic. **(e)** The embryo may initiate an endogenous “self-correction” program.

From the above findings, we observed that a SV existed in a mosaic state in the father and was transmitted to the offspring, who also exhibited mosaicism. According to classical genetic theory, if the father is a mosaic, his sperm population should theoretically consist of two types: one carrying a normal chromosome 11, and another carrying the abnormal chromosome 11 with an inserted SV. Given that this case involved a singleton pregnancy, the embryo presumably arose from the union of a single sperm and oocyte. Under typical genetic pattern, the offspring would be expected to present a non-mosaic normal karyotype (46,XX) or a non-mosaic abnormal karyotype 46,XX, ins (11;?) (p11;?) (Figure 4a). However, both prenatal AF and postnatal CB analyses revealed a high-level mosaic karyotype (Figure 4b), indicating that the embryo may have undergone an atypical chromosomal rearrangement during early development.

We hypothesize that the fetal mosaic state could have originated from homologous recombination events involving the short arms of sister chromatids during early mitotic divisions within the blastocyst’s inner cell mass. This process may have led to partial fragmentation and loss of the inserted SV, resulting in some cells reverting to a normal karyotype and others retaining the abnormal one, ultimately producing a mosaic state (Figures 4c,d). Supporting evidence for this hypothesis includes the presence of low-level cell populations in both prenatal amniotic fluid and postnatal cord blood showing 11p deletions or 11q duplications, suggesting that chromosomal fragments left unrepaired after the initial breakage remained in a small subset of cells.

#### 3.3.2 Literature summary of SMCs/SVs formation mechanisms

**FIGURE 5:**
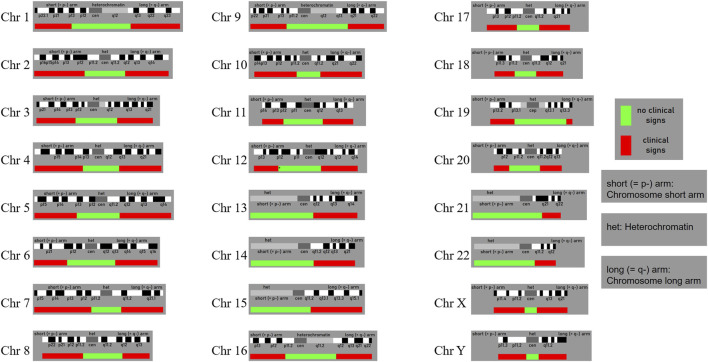
The probably non-dosage-sensitive pericentric regions of chromosomes 1 to 22 and the X and Y chromo-somes are shown in green. Dosage changes in these regions are unlikely to result in pathological clinical phenoty-pes. In contrast, regions shown in red indicate areas where dosage alterations are more likely to lead to abnormal clinical phenotypes. (Liehr T. 2025. Small supernumerary marker chromosomes. https://cs-tl.de/DB/CA/sSMC/0-Start.html).

Building on the findings of this study, we conducted an extensive literature search and database analysis to examine genetic patterns analogous to our case. Of particular note is the research led by Professor Liehr T, whose team has long focused on SMCs and established a globally recognized SMC database (Liehr T. 2025. Small supernumerary marker chromosomes. https://cs-tl.de/DB/CA/sSMC/0-Start.html, updated on 19 January 2025) (sSMC, 2025). This database incorporates numerous SMC cases involving autosomes 1–22 as well as the X and Y chromosomes, encompassing a variety of mosaic types and clinical phenotypes. Statistical data indicate that approximately 70% of SMC carriers are clinically asymptomatic, while the remaining 30% exhibit mild to severe abnormalities. Moreover, based on the chromosomal location of SMCs, genomic regions have been classified as either “dosage-sensitive” or “dosage-insensitive.” When SMCs occur in dosage-insensitive regions, most carriers show no overt clinical anomalies; by contrast, SMCs involving dosage-sensitive regions are more likely to present with clinical manifestations. Figure 5 illustrates the boundaries and distribution of these two categories across different chromosomes.

On this basis, we integrated database information and relevant literature to identify representative cases in which the parent carried an SMC/SV that was transmitted to the offspring, who also exhibited mosaicism (Table 1). In these cases, the parent (or other family members) carried the SMC/SV in nearly 100% or in mosaic form, and their offspring likewise presented a mosaic karyotype. Notably, the majority of such offspring were clinically normal, consistent with our findings that both the parent and child were phenotypically unaffected.

**TABLE 1:** Documented intergenerational transmissions of SMCs/SVs in which the offspring exhibit mosaicism.

NO	Type of SMCs	Offspring		Parent		Origin	Mechanism of SMC formation	PMID/Source
		SMC Mosaic rate	Clinical phenotype	SMC carrier status	Clinical phenotype			
1	min (2)(:p11.1-q21.1:)	75%	Prenatal early pregnancy loss	90% Mosaic	Phenotypically normal	maternal	Not reported in tde publication	16276087 (Liehr et al., 2006)
2	r (3) (::p10→q13.1::)	41%	Phenotypically normal	33% Mosaic	Phenotypically normal	maternal	Not reported in the publication	11241494 (Anderlid et al., 2001)
3	r (3)(::p10→q12::)	56%	Normal autopsy post-termination	5%–6% Mosaic	Phenotypically normal	maternal	Not reported in the publication	11241494 (Anderlid et al., 2001)
4	r (8) (::p11.21→q11.1::)	86%	Phenotypically normal	100%	Phenotypically normal	maternal	Not reported in the publication	13680362 (Starke et al., 2003)
5	idic (22)+ der (22)	82%	Normal autopsy post-termination	4% Mosaic	Phenotypically normal	paternal	Not reported in the publication	13680362 (Starke et al., 2003)
6	+r (8)(p11; q12)	86%	Phenotypically normal	100%	Phenotypically normal	maternal	Not reported in the publication	11354630 (Nietzel et al., 2001)
7	min (9) (:p12→q11:)	38%	Unrecorded data	73% Mosaic	Phenotypically normal	maternal	Not reported in the publication	852232^3^ (Müller-Navia et al., 1995)
8	min (9)	80%	Phenotypically normal	70% Mosaic	Phenotypically normal	maternal	*	7767653 (James et al., 1995)
9	mar (10) (:p11.21→q11.1:)	49%	Phenotypically normal (This case has a sister with the same karyotype, 77% mosaic, and a normal phenotype)	61% Mosaic	Phenotypically normal	maternal	Not reported in the publication	26270802 (Sant et al., 2015)
10	r (11)	60%	The patient is 34 years old and has CREST syndrome (a subtype of systemic sclerosis)	59% Mosaic	At the age of 59, progre ssive systemic sclerosis began to dev- elop and progressed to complete CREST syndrome	paternal	Not reported in the publication	1582251 (Haaf et al., 1992)
11	min (12) (:p11.1→q12:)	77%	Phenotypically normal	25% Mosaic	Phenotypically normal	paternal	Not reported in the publication	** (sSMC, 2025)
12	min (12) (:p11.1→q11:)	42.5%	Phenotypically normal	100%	Phenotypically normal	maternal	Not reported in the publication	** (sSMC, 2025)
13	min (14) (pter→q1?0)	19%	Phenotypically normal	100%	Phenotypically normal	maternal	Not reported in the publication	7747772 (Gravholt and Friedrich, 1995)
14	mar (14)	57%	Phenotypically normal	100%	Phenotypically normal	maternal	*	7767653 (James et al., 1995)
15	inv dup (15) (q11)	96%	Phenotypically normal (In this case, the sister carries the same as the father and has no abnormal phenotype)	100%	Phenotypically normal	paternal	Not reported in the publication	** (sSMC, 2025)
16	inv dup (15)(q11.1)	98%	Phenotypically normal	Mosaic (proportion not recorded)	Phenotypically normal	maternal	Not reported in the publication	** (sSMC, 2025)
17	inv dup (15) (q11.1)	83%	Phenotypically normal	100%	No abnormal phenotype observed in family members	Familial	Not reported in the publication	** (sSMC, 2025)
18	min (15) (:p11.1→q11.1:)	80%	Phenotypically normal	100%	Phenotypically normal (The mother inherited it from the grandmother of this case, who shows no abnormal phenotype)	maternal	Not reported in the publication	** (sSMC, 2025)
19	r (15)(::p11.2→q13.1::)	78.7%	Phenotypically normal	10% Mosaic	Phenotypically normal	maternal	Not reported in the publication	23295254 (Liehr et al., 2013)
20	r (15)	72%	Phenotypically normal	100%	Phenotypically normal	maternal	Not reported in the publication	16762822 (Karaman et al., 2006)
21	mar (15)	65%	Phenotypically normal	15% Mosaic	Phenotypically normal	maternal	Not reported in the publication	3688018 (Kousseff et al., 1987)
22	mar (15)	45%	Phenotypically normal	100%	Phenotypically normal	paternal	Not reported in the publication	16900777 (Kolialexi et al., 2006)
23	min (16)(:p11.2→q11.2:)	50%	Phenotypically normal	100%	Phenotypically normal	maternal	Not reported in the publication	** (sSMC, 2025)
24	mar (16) (:p11.2→q12.1:)	60%	Phenotypically normal	100%	Phenotypically normal	maternal	Not reported in the publication	28871159 (Sun et al., 2017)
25	min (16) (:p11.1→q12.1:)	20%	Phenotypically normal (This case is one of a twin pregnancy; the other fetus has no chromosomal abnormalities)	50% Mosaic	Phenotypically normal	maternal	Not reported in the publication	** (sSMC, 2025)
26	min (16) (:p11.1→q11.1:)	90%–97%	Phenotypically normal	4%–10% Mosaic	Phenotypically normal	paternal	Not reported in the publication	(Lee et al., 2009)
27	min (18) (:p11.21→q11.1:)	74%	Phenotypically normal	26% Mosaic	Phenotypically normal	maternal	Not reported in the publication	17317954 (Backx et al., 2007)
28	mar (18)(:p11.21→q11.1:)	80%	Phenotypically normal (This case gave birth to a daughter with the same SMC, who shows no abnormal phenotype)	100%	Phenotypically normal	paternal	Not reported in the publication	18252220 (Baldwin et al., 2008)
29	min (18) (:p11.1→q11.1:)	65%	Phenotypically normal	26% Mosaic	Phenotypically normal	maternal	Not reported in the publication	19816880 (Valentin et al., 2009)
30	mar (19) (:p12→q13.11:)	41%	Phenotypically normal	100%	Phenotypically normal	maternal	Not reported in the publication	39026136 (Jiang et al., 2024)
31	r (21)(::p1?2→q11.2::)	30%	Phenotypically normal	100%	Phenotypically normal	paternal	Not reported in the publication	** (sSMC, 2025)
32	inv dup (21) (q21.1)	47%	Phenotypically normal	22% Mosaic	Phenotypically normal	maternal	Not reported in the publication	32514314 (Zhou et al., 2020)
33	r (22) (::p12→q11.21::)	74%	Phenotypically normal	100%	Phenotypically normal	paternal	Not reported in the publication	33632263 (Liehr et al., 2021)
34	Min (X) (:p11.1→q11.1:)	70%	Phenotypically normal	100%	Phenotypically normal	maternal	Not reported in the publication	38259626 (Joksic et al., 2023)

** indicates the case source: Liehr T. 2025. Small supernumerary marker chromosomes. https://cs-tl.de/DB/CA/sSMC/0-Start.html.

* indicates the hypothesized formation mechanisms [PMID, 7767653].

①The SMC forms prior to meiosis, and a gamete carrying this SMC fuses with a normal gamete. Subsequent replication of the normal homolog partially rescues monosomy. ②A zygote formed by two normal gametes undergoes chromosomal breakage and SMC formation; duplication of the remaining normal chromosome then corrects monosomy. ③A normal diploid zygote duplicates one homolog, transitioning into a transient state of trisomy. The extra chromosome is subsequently lost, while forming an SMC to restore a diploid state. This process may result in uniparental disomy (UPD). ④Nondisjunction during meiosis produces a trisomic zygote. Partial correction occurs through the formation of an SMC (via chromosomal breakage) and loss of the extra chromosome, potentially leading to UPD. ⑤A pre-existing familial SMC and nondisjunction act in concert. After a triploid gamete fuses with a normal gamete, one normal chromosome is randomly lost, which may lead to UPD.

Drawing on these observations, we propose a new genetic regulatory model—designated the “Shepherd Mechanism”—in which, during early embryonic development, particularly the transition from fertilized egg to blastocyst, the embryo may initiate an endogenous “self-correction” program. Through processes such as selective apoptosis, developmental elimination, or cellular competition, cells bearing SMCs/SVs are purged, while cells with normal karyotypes are preferentially preserved in the inner cell mass. We further hypothesize that sperm or oocytes may carry an “Epigenetic memory” signal derived from the parent, which becomes activated in the early embryo, guiding the cell population toward a harmless level of mosaicism (Figure 4e). In this study, the paternal SV mosaic ratio was 57%, while the fetal ratios in AF and CB were 38% and 45%, respectively, closely mirroring the paternal mosaic level. Meanwhile, in the 33 related cases we reviewed, 73% (24/33) showed offspring mosaic ratios lower than or comparable to that of the parent, offering additional evidence that mosaicism may be regulated below a pathogenic threshold during development.

### 3.4 Computational modeling provides robust evidence for the “Shepherd Mechanism” in selective intergenerational mosaic regulation

#### 3.4.1 Computational modeling reveals non-random mosaic transmission mechanisms

To assess the biological plausibility of a developmental developmental self-regulatory mechanism underlying the selective intergenerational retention of benign SMCs/SVs mosaicism, we applied four complementary computational models, each designed to capture a different dimension of selection dynamics under realistic biological constraints.

**FIGURE 6:**
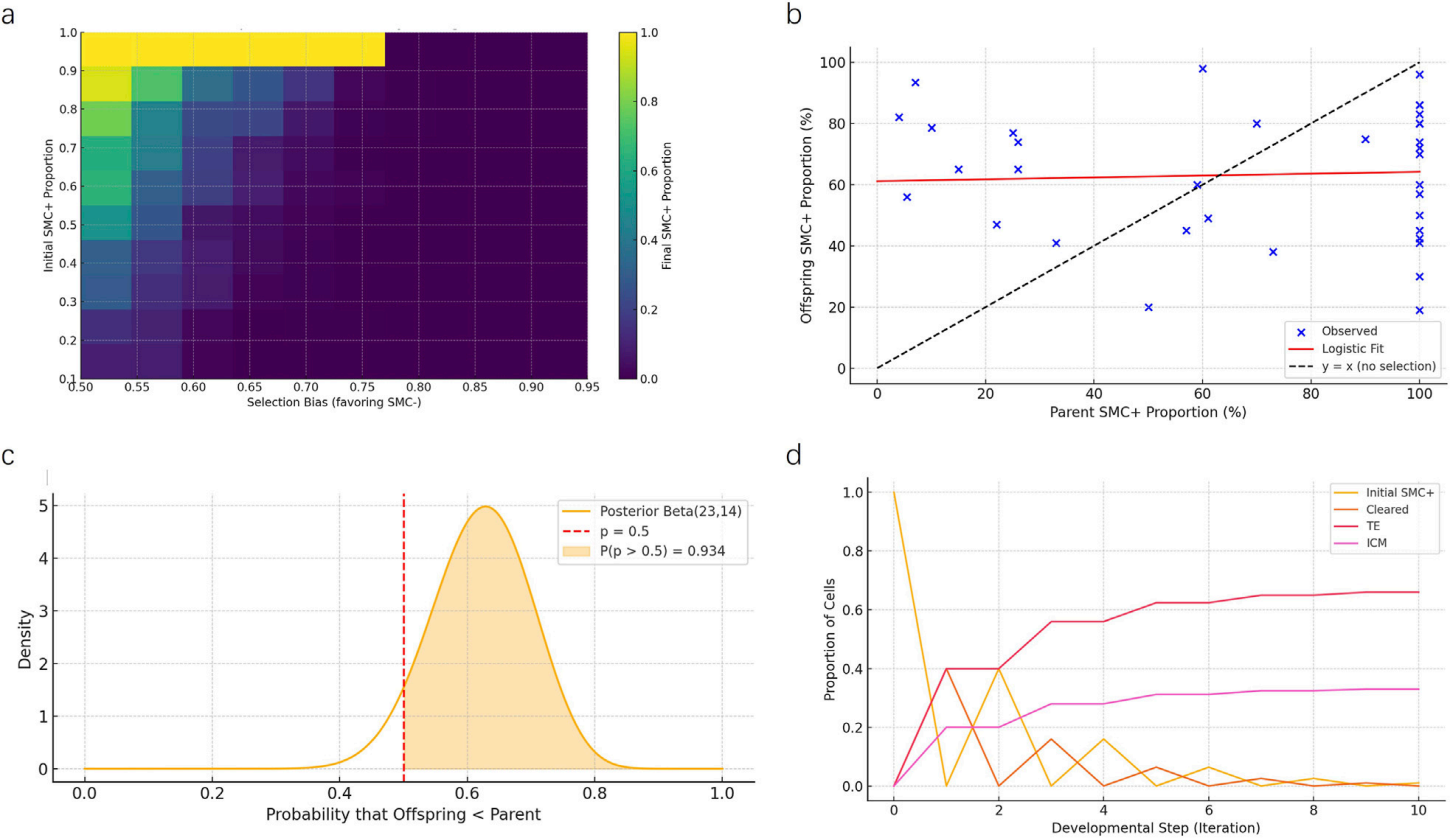
Multi-model computational evaluation of intergenerational mosaic retention mechanisms. **(a)** Heatmap from the agent-based simulation depicting final SMC/SV + cell proportions in the inner cell mass (ICM) under varying initial SMC/SV + levels and selection biases favoring SMC/SV–cells. Results indicate even moderate selection biases significantly reduce mosaic proportions. **(b)** Logistic regression modeling fitted to empirical data from 35 father-offspring mosaic cases. Observed data points (blue) deviate markedly from the random transmission expectation line (y = x, dashed line), highlighting selective moderation of mosaicism. **(c)** Bayesian posterior distribution analysis illustrates the probability that offspring mosaic levels are systematically lower than parental levels. The shaded region (P = 0.934) strongly rejects random inheritance. **(d)** Markov chain model showing developmental fate transitions of SMC/SV + cells over ten iterations. Most cells either undergo apoptosis (“Cleared”) or are preferentially allocated to the trophectoderm (TE), with a limited fraction retained in the ICM.

Using an agent-based modeling approach, we simulated how varying initial proportions of SMC/SV + cells and selection biases influenced their final retention levels within the inner cell mass (ICM). The heatmap illustrates that even mild selection biases favoring SMC/SV–cells markedly reduce the representation of SMC/SV + cells in the ICM, supporting the feasibility of a selective filtering mechanism during early embryogenesis (Figure 6a).

Logistic regression modeling was performed to quantitatively examine the relationship between parental and offspring mosaic ratios across 35 documented SMCs/SVs mosaic cases. The fitted logistic curve deviated substantially from the theoretical 1:1 random inheritance line (dashed line), confirming a non-linear, selection-mediated inheritance pattern and supporting the hypothesis that mosaic transmission is moderated rather than random (Figure 6b).

Bayesian posterior analysis assessed the likelihood that offspring mosaic proportions were systematically lower than parental proportions due to selective constraints. The posterior Beta distribution indicated a 93.4% probability that offspring mosaic levels were significantly lower than parental levels, thus strongly refuting the null hypothesis of random mosaic inheritance (Figure 6c).

Markov chain modeling of SMC/SV + cell fate transitions during developmental progression demonstrated rapid redistribution, with the majority of SMC/SV + cells cleared or allocated to the trophectoderm (TE). Only a minor fraction (∼33%) persisted in the ICM compartment after multiple developmental iterations, consistent with selective spatial compartmentalization (Figure 6d).

#### 3.4.2 Comparative evaluation of computational modeling approaches

To systematically assess and compare the strengths of each modeling strategy, we applied a structured evaluation framework based on four key criteria: mechanistic depth, spatial resolution, quantitative fit to empirical data, and statistical rigor (Table 2). The agent-based simulation provided the highest mechanistic depth and spatial resolution, accurately modeling cell-level interactions and tissue-specific compartmentalization, making it ideal for elucidating biological realism. Logistic regression exhibited superior quantitative accuracy, effectively capturing empirical trends of mosaic proportion inheritance. Bayesian inference excelled in statistical rigor, demonstrating strong quantitative support by calculating explicit posterior probabilities that robustly reject random inheritance scenarios. Markov chain analysis presented a balanced performance across all evaluation criteria, particularly excelling in dynamically representing developmental cell fate trajectories.

**TABLE 2:** Comparative evaluation of computational modeling approaches.

Model Type	Mechanistic Depth	Spatial Resolution	Quantitative Fit	Statistical Rigor
Agent-Based	High	High	Moderate	Moderate
Logistic	Low	None	High	Moderate
Bayesian	Low	None	Moderate	High
Markov	Moderate	Moderate	Moderate	Moderate

Qualitative scoring criteria (Low, Moderate, High) were adapted from established computational modeling evaluation frameworks, ensuring the rigor and reliability of these comparative assessments (Hodzic and Sindi, 2020; Developmental Biology; Grimm et al., 2020; Ecological Modelling).

#### 3.4.3 Computational evidence supporting the Shepherd Mechanism

The four computational models applied in this study, including agent-based simulation, logistic regression, Bayesian inference, and Markov-chain modeling, were not designed to uncover molecular or signaling pathways, but rather to evaluate, under realistic biological constraints, whether benign SMCs/SVs mosaicism could be stably maintained across generations. Each model approached this question from a distinct dimension, namely, cellular behavior, quantitative relationship, probabilistic assessment, and dynamic trajectory, and their convergent outcomes consistently demonstrated that intergenerational transmission of mosaic SMCs/SVs follows a self-moderating, non-random pattern within a benign range.

Collectively, these computational analyses revealed that mosaic cell populations tend to achieve a developmental equilibrium rather than random fluctuation, supporting the existence of a self-regulating developmental process that constrains mosaicism below a pathogenic threshold. The convergence of these independent modeling outcomes provides the theoretical basis for the proposed “Shepherd Mechanism”, a developmental self-regulation framework in which selective dynamics during early embryogenesis ensure stable, non-pathogenic inheritance of mosaicism across generations.

## 4 Discussion

Chromosomal mosaicism remains a challenging puzzle in genetics and reproductive medicine. Previous studies have shown that mosaic formation involves diverse mechanisms, including meiotic errors, chromosomal nondisjunction, and post-zygotic rescue events (McCoy, 2017; Grati, 2014; Levy et al., 2021). With the widespread use of preimplantation genetic testing (PGT) in in vitro fertilization (IVF), it has become evident that 3.1%–25% of human blastocysts may exhibit some degree of mosaicism (Popovic et al., 2020; Popovic et al., 2024). However, the clinical implications and appropriate management strategies for these mosaic embryos are still debated (Taylor et al., 2014).

### 4.1 Mosaic embryos in assisted reproduction

Our case demonstrates that a high level of mosaicism (57% in the father, 37%–45% in the offspring) can persist even under natural conception, aligning with the growing body of evidence on mosaic embryos in IVF contexts (Eggenhuizen et al., 2021; Coll et al., 2021; Viotti et al., 2021). In recent years, a central question in ART has been whether embryos exhibiting mosaicism should be transferred, especially when couples have limited embryos to choose from (Wang et al., 2024; Treff and Franasiak, 2017). Advanced techniques, such as deep sequencing and time-lapse imaging, have been employed to track chromosomal segregation and cellular competition in early-stage embryos (Sant et al., 2015). Some studies suggest that if the mosaic ratio is relatively low and does not involve dosage-sensitive regions, the embryo may retain significant developmental potential (Liehr and Al-Rikabi, 2019; Babariya et al., 2023). Nonetheless, data on mosaicism specifically caused by SMCs/SVs remain limited.

### 4.2 Further perspectives on the “Shepherd Mechanism”

Building on our concept of the “Shepherd Mechanism,” we hypothesize that embryos may possess a self-correction capacity during the blastocyst stage, leveraging processes like apoptosis and cellular competition to eliminate highly aberrant cells while preserving relatively normal cell lines in the inner cell mass (Popovic et al., 2020; Ma et al., 2017; Liehr et al., 2006). Recent high-resolution multi-omics studies suggest that three-dimensional chromatin architecture plays a pivotal role in cell fate determination (Perdigoto, 2017; Murphy et al., 2024). Abnormal chromatin topology can disrupt gene expression, potentially prompting the embryo to identify and remove such cells (Morales et al., 2020; Zheng and Xie, 2019). Intriguingly, some research indicates that the embryo may not eradicate all aberrant cells but instead maintain them below a “safe threshold,” allowing continued normal development (Yakovlev et al., 2022; Lin et al., 2020; Chen et al., 2021). In our case, the father and child both exhibited mosaic SV without any overt clinical manifestations, possibly due to this embryonic “quality control” mechanism (Ma et al., 2017; Zhong et al., 2023).

To further explore this hypothesis, we developed four computational models—agent-based simulation, logistic regression, Bayesian inference, and Markov chain modeling—to assess the developmental dynamics of SMC-bearing cell lineages. Each model provided distinct insights: agent-based simulations demonstrated that even weak selective pressures could reduce SMC + representation in the ICM; logistic regression revealed non-linear mosaic reduction trends; Bayesian inference showed high statistical support for reduced offspring mosaicism; and Markov modeling highlighted dynamic fate sorting of mosaic cells. While these findings provide convergent theoretical support for the Shepherd Mechanism, it is important to note that the modeling dataset comprised 35 literature-derived parent-offspring SMC mosaic cases. Although informative, this sample size may limit generalizability. Future validation with larger cohorts and empirical embryo data is necessary to substantiate these computational inferences. In recent years, studies using mouse chimeric embryo models have demonstrated that aneuploid cells undergo lineage-specific depletion around the blastocyst stage, and that mosaic embryos can develop into healthy individuals provided a sufficient proportion of euploid cells is present (Bolton et al., 2016). In both mouse and human early embryo and stem cell models, a cell competition mechanism operating from the blastocyst to peri-implantation stages mediates selective apoptosis and developmental selection, actively eliminating aneuploid or developmentally abnormal cells, thereby maintaining embryonic developmental homeostasis and self-correction capacity (Nichols et al., 2022).

### 4.3 Clinical practice and future directions

Clinically, understanding and validating such self-correction mechanisms is vital for refining PGT-A protocols. Recent studies involving multi-regional genomic analysis of trophectoderm and inner cell mass (Spinella et al., 2024) highlight that overestimating or underestimating the implantation potential of mosaic embryos can significantly impact embryo selection, particularly for couples with few viable options (Fragouli et al., 2019). Establishing clear screening criteria to distinguish “transferable, low-risk mosaic embryos” from “high-risk, potentially pathogenic mosaics” requires more robust prospective clinical trials and single-cell sequencing studies (Masset et al., 2022). On a mechanistic level, further studies are warranted to investigate how chromatin remodeling, epigenetic memory, and DNA damage response pathways interact during early development to influence the fate of mosaic cell populations. The formation and behavior of mosaics phenomena characterized by selectively established SMCs/SVs mosaic ratios remain particularly underexplored. Future directions should include *in vitro* validation using stem cell-derived embryo models, *in vivo* lineage tracing, and integration of multi-omics technologies (transcriptomics, epigenomics, 3D genomics) to dissect the molecular basis of mosaic resolution and persistence.

On a mechanistic level, elucidating how chromatin remodeling and genomic stability interact could provide deeper insights into the embryo’s capacity to identify and handle abnormal cells (Ma et al., 2017; Perdigoto, 2017). Detailed investigations into the breakpoints, duplicated regions, and functional consequences of SMCs/SVs may further substantiate the “Shepherd Mechanism” (Liehr and Al-Rikabi, 2019; Liehr et al., 2023). Moving forward, building advanced cell and embryo models and employing multi-dimensional omics (Transcriptomics, Epigenomics, 3D genomics) represent promising directions for future research.

In conclusion, the intergenerational transmission of mosaicism continues to pose significant challenges and opportunities in reproductive genetics. By integrating a naturally conceived case with existing literature, we propose a self-correction model that emphasizes early-stage cell selection and chromosomal remodeling. Validation of this concept in assisted reproduction and broader genetic counseling contexts could pave the way for more refined, personalized diagnostic and therapeutic strategies, offering more precise risk assessments and interventions for couples carrying chromosomal abnormalities.

## 5 Conclusion

Our integrative study provides compelling evidence supporting the existence of a novel regulatory mechanism, termed the “Shepherd Mechanism,” governing intergenerational mosaic SMCs/SVs transmission. Through cytogenetic analyses, comprehensive literature synthesis, and robust computational modeling, we demonstrate that embryonic development actively selects against mosaic-positive cells, effectively moderating mosaic proportions transmitted from parent to offspring. This selective regulation, validated by multiple computational approaches, represents a fundamental biological principle underpinning non-pathogenic mosaic inheritance. Our findings significantly advance current understanding of mosaic chromosome regulation and offer important insights for clinical genetic counseling and prenatal diagnosis.

## Data Availability

Data have been deposited in the NCBI GEO database. The GEO accession number is GSE302224, which allows for data tracking and public access in compliance with BMC Medical Genomics’ research data policy.
