# Supplementary material for: Benign mosaic chromosomal structural variants across generations: evidence for a developmental correction mechanism from clinical and computational models
Source: Front Genet. 2025 Nov 20;16:1710280. doi: 10.3389/fgene.2025.1710280 (PMC12676248; doi:10.3389/fgene.2025.1710280)
Supplement: Supplementary file 1 [file Table1.docx]

**CARE Checklist for the case report component**

| **Item** | **Description / Response** | **Notes** |
| --- | --- | --- |
| **1. Title** | The title indicates the focus on benign mosaic chromosomal variants across generations, corresponding to a case report integrated with modeling. | Tile |
| **2. Keywords** | Chromosomal mosaicism; Supernumerary marker chromosome (SMC); Structural variant chromosome (SV); Embryonic self-correction; Computational modeling. | KEY WORDS |
| **3. Abstract** | Structured summary including background, methods (case + literature + modeling), results (mosaic ratio), and conclusion (Shepherd Mechanism). | Section: Abstract |
| **4. Introduction** | Explains uniqueness of the case (father–offspring benign SMC/SV mosaicism) and establishes rationale. | Section 1 |
| **5. Patient Information** | De-identified: A 27-year-old woman (G3P0) and her husband; both healthy, referred due to paternal mosaic abnormality; ethical approval 2024-Y-66. | Section 2.1 |
| **6. Clinical Findings** | Prenatal and postnatal karyotyping and SNP-array showed mosaic SV at 11p11; no phenotypic abnormalities. | Sections 3.1–3.2 |
| **7. Timeline** | Timeline from genetic counseling → amniocentesis (18 weeks) → delivery → follow-up. | Figure/Table optional |
| **8. Diagnostic Assessment** | Karyotype (G-banding), SNP-array; interpretation under ACMG/ClinGen; compared AF, CB, and paternal samples. | Section 2.2.1 |
| **9. Therapeutic Intervention** | No therapeutic intervention; observational study only. | N/A |
| **10. Follow-up and Outcomes** | Child followed regularly, no developmental anomalies to date. | Section 2.1 |
| **11. Discussion** | Discusses biological rationale, literature context, modeling integration, and take-away lessons (Shepherd Mechanism). | Section 4 |
| **12. Patient Perspective** | N/A (scientific case without direct patient narrative). | N/A |
| **13. Informed Consent** | Written informed consent obtained; Ethics approval 2024-Y-66. | Section 2.1 |
