# Supplementary material for: Benign mosaic chromosomal structural variants across generations: evidence for a developmental correction mechanism from clinical and computational models
Source: Front Genet. 2025 Nov 20;16:1710280. doi: 10.3389/fgene.2025.1710280 (PMC12676248; doi:10.3389/fgene.2025.1710280)
Supplement: Supplementary file 2 [file Table2.docx]

**PRISMA 2020 Checklist for the systematic review component**

| **PRISMA 2020 Item** | **Section / Topic** | **Location in manuscript / Response** |
| --- | --- | --- |
| **1. Title** | Title page | Title indicates inclusion of clinical and computational evidence (includes literature review). |
| **2. Abstract** | Abstract | Summary of systematic review component and modeling integration. |
| **3. Rationale** | Introduction | Justifies review of benign SMCs/SVs inheritance mechanisms. |
| **4. Objectives** | Introduction | States goal: to elucidate intergenerational mosaic stability. |
| **5. Eligibility Criteria** | 2.2.2 | Included studies with explicit parent-offspring SMC/SV mosaicism; English only. |
| **6. Information Sources** | 2.2.2 | PubMed, Web of Science, Scopus. |
| **7. Search Strategy** | 2.2.2 | Keywords: “supernumerary marker chromosome,” “mosaicism,” “prenatal diagnosis,” “inheritance.” |
| **8. Selection Process** | 2.2.2 | Articles screened for explicit parent-offspring SMCs/SVs cases; excluded those without origin data. |
| **9. Data Collection Process** | 2.2.2 | Extracted mosaic ratios, SMC type, chromosomal regions, and clinical features. |
| **10. Data Items** | 3.3.2, Table 1 | Data include mosaic proportions, inheritance patterns, clinical phenotypes. |
| **11. Risk of Bias Assessment** | Discussion 4.2 | Potential publication bias acknowledged due to small sample size. |
| **12. Results of Studies** | Table 1 | Summarized 35 families with mosaic transmission. |
| **13. Synthesis of Results** | 3.3.2–3.4 | Integrated quantitative comparison and modeling-based interpretation. |
| **14. Discussion of Limitations** | 4.2 | Notes limited generalizability due to small number of cases. |
| **15. Funding** | Acknowledgment (if added) | Institutional research; no external commercial funding. |
| **16. Registration / Protocol** | N/A | Not pre-registered (retrospective synthesis). |
